# Diabetes ketoacidosis in L-asparaginase therapy

**DOI:** 10.1186/1687-9856-2013-S1-P21

**Published:** 2013-10-03

**Authors:** Marichu P Mabulac, Carol Boongaling, Lorna R Abad

**Affiliations:** 1 Section of Pediatric Endocrinology, Department of Pediatrics, University of the Philippines-Philippine General Hospital, Manila, Philippines

## Background

Diabetic ketoacidosis as a complication of L asparaginase therapy in children with acute leukemia is rare. We report a patient with acute lymphoblastic leukemia who developed diabetic ketoacidosis while on treatment with L-asparaginase.

## Case

E.C, 10 years old female brought to the ER due abdominal pain. Patient is a diagnosed case of B-Cell Acute Lymphoblastic Leukemia (ALL) thru clinical symptoms, laboratory work-ups and flow cytometry since 2 months prior to her present admission. She has been receiving 8 doses of L-asparaginase (leunase) and Prednisone (60mg/day for a week then 40mg/day for 3 weeks) for almost one month before this recent admission. One week prior to hospitalization, the patient had nocturia (2-3x/night), polyuria and polydipsia. On the day of admission, she had sudden onset difficulty of breathing associated with severe abdominal pain and vomiting. She came in non-ambulatory, in cardiorespiratory distress with BP: 100/70mmHg, HR: 150/min, RR: 60/min, Temp: 37.3°C. Pertinent physical exam showed signs of dehydration with abdominal tenderness, and signs of circulatory compromise. Fluid resuscitation was immediately instituted. The initial assessment at the ER was pancreatitis but the serum amylase and lipase were normal. Other lab work-ups showed blood glucose of 26.8 mmol/L (NV:3.08-7.92), sodium at 115 mmol/L(NV:132-143), potassium of 3.3, Chloride at 88mmol/L (NV: 98-116). Blood gas showed ph of 7.1, a pCO2: 50, HCO3 at 3.7, and a base deficit of -22.5mmol/L. Urinalysis showed +4 of glucose and +3 of ketones. The patient was then managed as a case of DKA and fluid resuscitation was continued and insulin drip was started. The haemoglobin A1c level at diagnosis was 11.4% but the C-peptide was normal. Patient condition improved and was discharged with insulin injection. The insulin was discontinued after completion of her induction phase and with normalization of blood glucose.

## Conclusion

Early recognition of the precipitating factors for DKA is important to prevent L-asparaginase fatal consequences, and the leukemic process itself maybe considered as one of the predisposing factors.

